# Supplementary material for: RootNav 2.0: Deep learning for automatic navigation of complex plant root architectures
Source: Gigascience. 2019 Nov 8;8(11):giz123. doi: 10.1093/gigascience/giz123 (PMC6839032; doi:10.1093/gigascience/giz123)
Supplement: giz123_Response_to_Reviewer_Comments_Original_Submission [file giz123_response_to_reviewer_comments_original_submission.pdf]

Author's Response To Reviewer Comments

Close

Many thanks for your swift handling of our paper. We hope we have addressed all of the concerns to your, and the reviewers, satisfaction. We have updated the software to address concerns raised about long term support, and have outlined a plan to ensure the tool continues to be supported in the long term. We have also added comments, additional supplementary figures and new manuscript text addressing the remaining reviewer comments. Finally, the tool is now tracked on SciCrunch at RRID SCR_015584. Changes to the manuscript are highlighted in red. We include a response to reviewers below, but please find a formatted version among the submitted files for convenience.

We thank the reviewers for their critical assessment of our work. In the following we respond to each concern on a point by point basis. We have highlighted changes to the manuscript in red text.

REVIEWER #1

Comment: RootNav 2.0 requires Python 2.7, which will not be supported beyond 2020. As reuse is a major objective of GigaScience, I invite the authors to provide a detailed plan of how they will ensure continued support for RootNav 2.0 software in the longer term. For long-term reusability, the authors should consider updating the RootNav 2.0 code so that it utilises a version of Python 3 that will be supported long term.

Reply: The reviewer is absolutely right to raise this point. Reliance on Python 2 is not conducive to the long term use of the tool by the community. We have now fully updated the tool to be primarily Python 3 based (currently Python 3.6), and have removed and improved many of the dependencies to simplify the installation process. We have tested this new version on all three major operating systems and found that it works effectively. We have updated the source code and requirements section of the manuscript.

Over the longer term, we will continue to support this tool in the following ways:

- We are exploring collaborations to broaden the number of available models for different species and imaging types.
- We have introduced a "model history" feature that allows anyone contributing a new model to receive appropriate credit for their contribution, as well as those whose models are used to retrain new ones. New models are downloaded automatically by the tool as they become available and are needed.
- As RootNav 2.0 is now open source, we will support any contribution from the community including code improvements and features.
- We will explore the development of a client server architecture where the bulk of the processing may be performed on a more powerful remote machine. This will simplify installation where multiple devices can access a single RootNav 2.0 installation, rather than all have unique copies. Part of this functionality is already implemented.
- We are commissioning a redesign of plantimages.nottingham.ac.uk to support larger numbers of datasets and external contributors. We will also obtain a permanent DOI for this resource. This will include links to the relevant software tools such as RootNav 2.0.

If acceptable to the reviewers and editor we have added an acknowledgement to Dr. Michael Wilson, who aided in the testing of the multi-platform deployment of the tool as we made the above changes.

Comment: Filenames for tabular data are not unique. For example, output/ and plots/data/ directories include files named arabidopsis.csv and wheat.csv. Unique filenames are less ambiguous and the authors should consider revising the filenames.

Reply: We have altered the file names to avoid these duplicates.

Comment: The tabular data file plots/data/arabidopsis.csv has a column with no header information.

Please add header information to this file.

Reply: Thank you. This column was added in error and not was not required for the plot script, we have removed it.

Comment: The tabular data file output/arabidopsis.csv has additional columns named Plant ID, Absolute X, and Absolute Y that are not found in the tabular data output files for wheat and rapeseed analysis. I invite the authors to clarify the differences between the output files.

Reply: The Arabidopsis output differs slightly from the other two as it contains multiple plants per image. The PlantID is meaningful here as it tracks plants within an image, but is not used in the plot output. We used absolute X and Y positions as guidance to identify incorrect plants. These columns were used by ourselves to identify correctly and incorrectly located plants (Figure 7, Plant count accuracy), but were not used in accuracy measurements in the other plots.

The RootNav Viewer tool (found in the github repository) allows many other measurements to be captured from RSML files, these can be selected and deselected as desired. Thus these additional measurements could be captured for other datasets as well if helpful for any new experiments.

REVIEWER #2

Comment: More extensive documentation is not currently available, though the README is sufficiently detailed to install and run the program

Reply: We have updated the README file to include more detailed installation and usage instructions, and simplified the command line interface to the training and inference parts of the tool to also make this easier. We will continue to improve the training part of the tool over time, which we see as the most complex of the two.

Typographical errors

* Page 3: "...and may cause produce additional false positives where the small field of view is not sufficient to distinguish true roots from image noise." Remove the word "cause".

* On Pages 4 and 7, replace "infra red" with "infrared". Both are correct but I think it's more common for it to be one word.

* Page 12: "In the case of RSA traversal, minimal cost paths between key features such first order root tips and seeds represent reconstructed roots." Insert the word "as" between "such first".

Reply: We have corrected these typographical errors, thank you.

REVIEWER #3

Comment: How well will this model perform for more mature root structures, beyond simple assays where the roots are essentially 2-D? As mentioned in Ref. [42] that Hourglass network performs poor with occlusion; is this problem addressed by A* algorithm for a mature plant with dense roots that form a 3D structure?

Reply: In [42] the hourglass network is used exclusively for heatmap regression, the component of our network that finds the root tips and seed locations. For very dense root systems, or roots whose tips are very close together, it is likely that the number of tips detected is lower than the ground truth. We utilise non-maximal suppression to avoid duplicate tips, but this has the effect of also removing tips that lie very close together. This can be observed in plot results, where for first and second order tip counting, each line of best fit is slightly below 1*x, indicating a slight underestimate of count. The segmentation and path finding components of the algorithm will be unaffected, working similarly on dense or sparse data. However, path finding may join roots that are close together if the edges of each root are not well separated, where one of a number of roots is judged to be an optimal path. This will add some noise to measurements such as total root length, but is unlikely to affect measurements of the boundary of the root system. Examples may be seen in the new Supplementary Figure 9.

Comment: The very small dataset for Arabidopsis (200 training image) and rapeseed (91) can induce

overfitting in the network and the sample result is not shown or discussed. This needs clarification.

Reply: We have added two supplementary figures (Supplementary Figures 8 and 9) containing clearer examples of overfitting to illustrate the drawbacks of smaller dataset sizes. These are test set images, and so quantitative results from these are already included in our results. We discuss overfitting in the manuscript, and the drop in accuracy caused by it. We also mention that we utilise transfer learning to improve performance specifically for this reason (E.g. Results, Arabidopsis thaliana). We have added additional text to the manuscript results drawing attention to the effect of overfitting, and referencing the new supplementary figures.

Comment: As per Fig.4, Arabidopsis sample image has 5 root structures in a single frame. The heatmap regression will detect 5 seeds. How does the navigation algorithm work between different root tips detected and seeds? When does this not work? It would also help to see sample test results comparing the before and after images.

Reply: Thank you for noticing that we did not mention this in the methods - this was an oversight on our part. Path traversal works in a similar way for multiple plants. Firstly, a meaningful remaining distance heuristic is difficult and inefficient to calculate where multiple goals exist, so we utilise Dijkstra's algorithm rather than A* - this is also true of second order root paths.

The search for multiple plants then proceeds as follows: A plant is created at each seed location, initially assuming all seed locations are correct. Each first order tip location begins a search back to any available plant. The first plant reached is the shortest path, and that root path is assigned to that plant. This process is repeated for all first order roots.

Any plants that are not assigned at least one root once this process is completed are removed. This approach is robust to many instances of incorrect seed locations, which are simply ignored if no suitable first order root is found. The process is not robust to incorrect primary root tips, which will be connected with a seed unless the path length is unusually long. We have added text to the results and methods sections outlining this process in more detail.

Comment: If the dataset is a single channel (no colour information) model compared to the 3 channel wheat model, would any modifications need to be made to the network to include this change?

Reply: Thank you for querying this important issue. Normally the first layer of a network should indeed be adapted to handle a different number of input channels. However to ensure compatibility between models, in particular to simplify transfer learning between images that may move between RGB and single channel, we chose to fix the network to use three input channels in all cases. For the Arabidopsis dataset, we duplicate the grayscale channels into the RGB channels prior to use. The computational overhead of this only affects the weights in the first layer of a very deep network, and is marginal. The result is that the Arabidopsis model does not make use of channel information in the first layer, but remains structurally identical to the other two models. We have added this information into the manuscript (Methods, Transfer Learning).

Comment: Table 3: comparison between RootNav 1 and 2 seems a little odd, i.e. trying to compare time w.r.t human intervention and full automation. Especially w.r.t Avg processing time, which is highly dependent on features. A new table should be created specific to one crop (you can have 1 table per crop or just stick to any one crop). The new table 3 should be augmented with additional rows and columns as following: Columns = accuracy (rootnav 1 vs 2); rows = coarse features (a. convex hull, b. primary root length c. plant count etc) ; fine features (d. secondary root count, e. secondary lengths etc)

Reply: We presented a timing comparison as a coarse demonstration of the speed at which RootNav 2.0 typically operates compared to the original tool. Accuracy is compared on both fine and course traits in Figures 6-8. We agree that average timing measures are an oversimplification in the case where a user is not measuring finer detailed features. For example, should the user require manual measurement of the primary root length only, this would require much less time on the rapeseed dataset than full annotation of the second order roots. However, many of the features we might initially identify as coarse, such as convex hull, actually require the detection of second order root tips and paths, and so inevitably a full RSA reconstruction. The measured time taken by both tools is almost entirely in the RSA extraction process, while the actual measurement of traits from RSML is almost instantaneous, regardless of which trait. We wanted to present a like-for-like comparison, showing each tool when used

to extract as many traits of interest as possible, particularly where these traits may be used for gene discovery, so pertinent phenotypes are not yet known. We have added text to the manuscript to make clear the nature of this test, and that RootNav 1.0 may be used for faster analysis where only a small subset of traits are required.

Comment: The r^2 values seem to indicate that coarse features (overall length, convex areas) are extracted accurately, whereas the fine features (root counts, second order root features) are still challenging even with RootNav2. In that sense it still has ways to go before it can replace semi-automation. What magnitude of statistical errors can a GWAS handle and is the error output of RootNav2 within those bounds? Some discussion on this needs to be made. And maybe it should be made clear that further improvements can be anticipated in a RootNav 3?

Reply: We agree that the fully-automatic approach offered in RootNav 2.0 does not achieve the same accuracy as a semi-automatic method on some measurements. This is assuming an expert user who has taken sufficient time over the semi-automatic measurement. This is reflected in the results presented in this paper. We believe that the speed of RootNav 2.0 will allow much higher throughout analysis, which will actually result in *more* reliable GWAS, rather than less. We have added discussion on this point within the discussion section of the manuscript including relevant citations. [46] and [47] show that higher sample sizes for QTL and GWAS improve performance, as they permit higher numbers of replicates. We have also shown in our previous work [29] that even simple features drawn only from root tips were able to find similarly strong and even new QTLs when compared to semi-automatic measures. Nevertheless, we will continue to strive towards the highest accuracy, and we now note this also in the discussion section of the manuscript.

Close
