# Supplementary material for: RootNav 2.0: Deep learning for automatic navigation of complex plant root architectures
Source: Gigascience. 2019 Nov 8;8(11):giz123. doi: 10.1093/gigascience/giz123 (PMC6839032; doi:10.1093/gigascience/giz123)
Supplement: giz123_Response_to_Reviewer_Comments_Revision_1 [file giz123_response_to_reviewer_comments_revision_1.pdf]

Author's Response To Reviewer Comments

Close

Many thanks for the swift processing of this manuscript. I have corrected the manuscript as per the last editor's comments. I have also removed the red highlights from the text indicating previous changes. The tool now has appropriate biotools and scicrunch identifiers included.

Close
