# Supplementary material for: RootNav 2.0: Deep learning for automatic navigation of complex plant root architectures
Source: Gigascience. 2019 Nov 8;8(11):giz123. doi: 10.1093/gigascience/giz123 (PMC6839032; doi:10.1093/gigascience/giz123)
Supplement: giz123_Reviewer_1_Report_Original_Submission — Chris Armit -- 7/29/2019 Reviewed [file giz123_reviewer_1_report_original_submission.pdf]

Reviewer Report

Title: RootNav 2.0: Deep Learning for Automatic Navigation of Complex Plant Root Architectures

Version: Original Submission **Date:** 7/29/2019

Reviewer name: Chris Armit

Reviewer Comments to Author:

In this Research article the authors report on RootNav 2.0, which is image analysis software that utilises Convolutional Neural Network (CNN) architecture to provide fully-automated extraction of complex root system morphology from images from a range of plant species. The manuscript is well-written and the authors are to be commended for providing a detailed explanation of the problems associated with segmenting 2D projection images (Figure 1), and for providing details of how RootNav 2.0 overcomes these issues and performs superior to other commonly used CNN architectures (Figure 5). RootNav 2.0 is proficient at segmenting first order roots, second order roots, and second order tip locations. A key feature of the RootNav 2.0 image analysis process is that roots are re-sampled as smooth splines, with spline fitting utilising distance maps that prioritise the centre line of roots. RootNav 2.0 is available on GitHub with an OSI-approved 3-Clause BSD license, which ensures that the software are open and accessible. The image data and output files, including masks, have been submitted to GigaDB.

Major issue

RootNav 2.0 requires Python 2.7, which will not be supported beyond 2020. More details about this are available at the following link: https://pythonclock.org/

As reuse is a major objective of GigaScience, I invite the authors to provide a detailed plan of how they will ensure continued support for RootNav 2.0 software in the longer term. For long-term reusability, the authors should consider updating the RootNav 2.0 code so that it utilises a version of Python 3 that will be supported long term.

Minor issues

1. Filenames for tabular data are not unique. For example, output/ and plots/data/ directories include files named arabidopsis.csv and wheat.csv. Unique filenames are less ambiguous and the authors should consider revising the filenames.
2. The tabular data file plots/data/arabidopsis.csv has a column with no header information. Please add header information to this file.
3. The tabular data file output/arabidopsis.csv has additional columns named Plant ID, Absolute X, and Absolute Y that are not found in the tabular data output files for wheat and rapeseed analysis. I invite the authors to clarify the differences between the output files.

Methods

Are the methods appropriate to the aims of the study, are they well described, and are necessary controls included? Choose an item.

Conclusions

Are the conclusions adequately supported by the data shown? Choose an item.

Reporting Standards

Does the manuscript adhere to the journal's guidelines on minimum standards of reporting? Choose an item.

Choose an item.

Statistics

Are you able to assess all statistics in the manuscript, including the appropriateness of statistical tests used? Choose an item.

Quality of Written English

Please indicate the quality of language in the manuscript: Choose an item.

Declaration of Competing Interests

Please complete a declaration of competing interests, considering the following questions:

- Have you in the past five years received reimbursements, fees, funding, or salary from an organisation that may in any way gain or lose financially from the publication of this manuscript, either now or in the future?
- Do you hold any stocks or shares in an organisation that may in any way gain or lose financially from the publication of this manuscript, either now or in the future?
- Do you hold or are you currently applying for any patents relating to the content of the manuscript?
- Have you received reimbursements, fees, funding, or salary from an organization that holds or has applied for patents relating to the content of the manuscript?
- Do you have any other financial competing interests?
- Do you have any non-financial competing interests in relation to this paper?

If you can answer no to all of the above, write 'I declare that I have no competing interests' below. If your reply is yes to any, please give details below.

I declare that I have no competing interests.

I agree to the open peer review policy of the journal. I understand that my name will be included on my report to the authors and, if the manuscript is accepted for publication, my named report including any attachments I upload will be posted on the website along with the authors' responses. I agree for my

report to be made available under an Open Access Creative Commons CC-BY license (http://creativecommons.org/licenses/by/4.0/). I understand that any comments which I do not wish to be included in my named report can be included as confidential comments to the editors, which will not be published.

Choose an item.

To further support our reviewers, we have joined with Publons, where you can gain additional credit to further highlight your hard work (see: https://publons.com/journal/530/gigascience). On publication of this paper, your review will be automatically added to Publons, you can then choose whether or not to claim your Publons credit. I understand this statement.

Yes Choose an item.
