# Supplementary material for: RootNav 2.0: Deep learning for automatic navigation of complex plant root architectures
Source: Gigascience. 2019 Nov 8;8(11):giz123. doi: 10.1093/gigascience/giz123 (PMC6839032; doi:10.1093/gigascience/giz123)
Supplement: giz123_Reviewer_2_Report_Original_Submission — Noah Fahlgren -- 8/8/2019 Reviewed [file giz123_reviewer_2_report_original_submission.pdf]

Reviewer Report

Title: RootNav 2.0: Deep Learning for Automatic Navigation of Complex Plant Root Architectures

Version: Original Submission **Date:** 8/8/2019

Reviewer name: Noah Fahlgren

Reviewer Comments to Author:

In the manuscript "RootNav 2.0: Deep Learning for Automatic Navigation of Complex Plant Root Architectures" by Yasrab and colleagues, a new tool, RootNav 2.0, is presented. Unlike the semi-automated RootNav precursor tool, RootNav 2.0 uses a deep learning-based approach to eliminate the human-in-the-loop steps required to reconstruct root system architecture in the original RootNav. RootNav 2.0 uses an encoder-decoder-based convolutional neural network architecture to learn to predict the location of seeds and root tips and to segment primary and secondary roots from 2D root images. The segmented root features are then used to reconstruct the root system architecture using a class-aware (primary or secondary) pathfinding algorithm. The reconstructed root system architecture is output in the widely used Root System Markup Language standard format for use with downstream tools. The authors provide three pretrained models with RootNav 2.0 and demonstrate that new models can be generated with relatively minimal training data by building on one of the existing models (transfer learning). RootNav 2.0 segmentation accuracy was very high on unseen data and performed well on measuring root system architecture traits compared to expert labeled data.

Strengths

- The deep learning-based root segmentation approach used here is unique and effective.
- The use of a customized network that minimizes the number of trainable parameters to keep the network memory usage small, and therefore keep image sizes large is a clever idea. This is a common problem for using deep learning for plant phenotyping because images tend to be large and plant features tend to be small/thin and downsizing the images to fit within memory constraints tends to lose plant information.
- Using the semi-automated RootNav tool to create training data for the automated RootNav 2.0 tool is a nice way to help users build training datasets in an efficient manner.
- Segmenting roots into primary and secondary classes and using this information to improve pathfinding is a great idea.
- The manuscript is well-written and accessible.
- The code and pretrained models are freely available on GitHub under a permissive license. Data used in the study is available for download. Reproducing the results in the manuscript was straightforward.
- Installation and use of RootNav 2.0 was straightforward and is well-documented in the GitHub README file.

Weaknesses

- More extensive documentation is not currently available, though the README is sufficiently detailed to install and run the program.

Overall, this is an excellent manuscript and a useful tool. I have a few minor grammatical suggestions:

- Page 3: "...and may cause produce additional false positives where the small field of view is not sufficient to distinguish true roots from image noise." Remove the word "cause"
- On Pages 4 and 7, replace "infra red" with "infrared". Both are correct but I think it's more common for it to be one word.
- Page 12: "In the case of RSA traversal, minimal cost paths between key features such first order root tips and seeds represent reconstructed roots." Insert the word "as" between "such first"

Methods

Are the methods appropriate to the aims of the study, are they well described, and are necessary controls included? Choose an item.

Conclusions

Are the conclusions adequately supported by the data shown? Choose an item.

Reporting Standards

Does the manuscript adhere to the journal's guidelines on minimum standards of reporting? Choose an item.

Choose an item.

Statistics

Are you able to assess all statistics in the manuscript, including the appropriateness of statistical tests used? Choose an item.

Quality of Written English

Please indicate the quality of language in the manuscript: Choose an item.

Declaration of Competing Interests

Please complete a declaration of competing interests, considering the following questions:

- Have you in the past five years received reimbursements, fees, funding, or salary from an organisation that may in any way gain or lose financially from the publication of this manuscript, either now or in the future?
- Do you hold any stocks or shares in an organisation that may in any way gain or lose financially from the publication of this manuscript, either now or in the future?
- Do you hold or are you currently applying for any patents relating to the content of the manuscript?
- Have you received reimbursements, fees, funding, or salary from an organization that holds or has applied for patents relating to the content of the manuscript?

- Do you have any other financial competing interests?
- Do you have any non-financial competing interests in relation to this paper?

If you can answer no to all of the above, write 'I declare that I have no competing interests' below. If your reply is yes to any, please give details below.

I declare that I have no competing interests

I agree to the open peer review policy of the journal. I understand that my name will be included on my report to the authors and, if the manuscript is accepted for publication, my named report including any attachments I upload will be posted on the website along with the authors' responses. I agree for my report to be made available under an Open Access Creative Commons CC-BY license (http://creativecommons.org/licenses/by/4.0/). I understand that any comments which I do not wish to be included in my named report can be included as confidential comments to the editors, which will not be published.

Choose an item.

To further support our reviewers, we have joined with Publons, where you can gain additional credit to further highlight your hard work (see: https://publons.com/journal/530/gigascience). On publication of this paper, your review will be automatically added to Publons, you can then choose whether or not to claim your Publons credit. I understand this statement.

Yes Choose an item.
