# Supplementary material for: RootNav 2.0: Deep learning for automatic navigation of complex plant root architectures
Source: Gigascience. 2019 Nov 8;8(11):giz123. doi: 10.1093/gigascience/giz123 (PMC6839032; doi:10.1093/gigascience/giz123)
Supplement: giz123_Reviewer_3_Report_Original_Submission — Srikant Srinivasan -- 8/19/2019 Reviewed [file giz123_reviewer_3_report_original_submission.pdf]

Reviewer Report

Title: RootNav 2.0: Deep Learning for Automatic Navigation of Complex Plant Root Architectures

Version: Original Submission **Date:** 8/19/2019

Reviewer name: Srikant Srinivasan

Reviewer Comments to Author:

The advances in plant breeding and computational techniques of GWAS has spurred automation in high throughput plant phenotyping but most advances have been in traits that are above ground. Root phenotyping is recognized as an area that would benefit from full automation. As such the work presented in this paper is very relevant to the current needs of the plant phenotyping community and the authors have written it in a manner that would be useful for others. The RootNav 2.0 architecture is unique as from the same encoder-decoder both segmentation and localization are performed. The following clarifications will help strengthen the paper:

1. How well will this model perform for more mature root structures, beyond simple assays where the roots are essentially 2-D? As mentioned in Ref. [42] that Hourglass network performs poor with occlusion; is this problem addressed by A* algorithm for a mature plant with dense roots that form a 3D structure?
2. The very small dataset for Arabidopsis (200 training image) and rapeseed (91) can induce overfitting in the network and the sample result is not shown or discussed. This needs clarification.
3. As per Fig.4, Arabidopsis sample image has 5 root structures in a single frame. The heatmap regression will detect 5 seeds. How does the navigation algorithm work between different root tips detected and seeds? When does this not work? It would also help to see sample test results comparing the before and after images.
4. If the dataset is a single channel (no colour information) model compared to the 3 channel wheat model, would any modifications need to be made to the network to include this change?
5. Table 3: comparison between RootNav 1 and 2 seems a little odd, i.e. trying to compare time w.r.t human intervention and full automation. Especially w.r.t Avg processing time, which is highly dependent on features. A new table should be created specific to one crop (you can have 1 table per crop or just stick to any one crop). The new table 3 should be augmented with additional rows and columns as following: Columns = accuracy (rootnav 1 vs 2); rows = coarse features (a. convex hull, b. primary root length c. plant count etc) ; fine features (d. secondary root count, e. secondary lengths etc)
6. Lastly and most importantly, the r^2 values seem to indicate that coarse features (overall length, convex areas) are extracted accurately, whereas the fine features (root counts, second order root features) are still challenging even with RootNav2. In that sense it still has ways to go before it can replace semi-automation. What magnitude of statistical errors can a GWAS handle and is the error output of RootNav2 within those bounds? Some discussion on this needs to be made. And maybe it should be made clear that further improvements can be anticipated in a RootNav 3?

Methods

Are the methods appropriate to the aims of the study, are they well described, and are necessary controls included? Choose an item.

Conclusions

Are the conclusions adequately supported by the data shown? Choose an item.

Reporting Standards

Does the manuscript adhere to the journal's guidelines on minimum standards of reporting? Choose an item.

Choose an item.

Statistics

Are you able to assess all statistics in the manuscript, including the appropriateness of statistical tests used? Choose an item.

Quality of Written English

Please indicate the quality of language in the manuscript: Choose an item.

Declaration of Competing Interests

Please complete a declaration of competing interests, considering the following questions:

- Have you in the past five years received reimbursements, fees, funding, or salary from an organisation that may in any way gain or lose financially from the publication of this manuscript, either now or in the future?
- Do you hold any stocks or shares in an organisation that may in any way gain or lose financially from the publication of this manuscript, either now or in the future?
- Do you hold or are you currently applying for any patents relating to the content of the manuscript?
- Have you received reimbursements, fees, funding, or salary from an organization that holds or has applied for patents relating to the content of the manuscript?
- Do you have any other financial competing interests?
- Do you have any non-financial competing interests in relation to this paper?

If you can answer no to all of the above, write 'I declare that I have no competing interests' below. If your reply is yes to any, please give details below.

I declare that I have no competing interests

I agree to the open peer review policy of the journal. I understand that my name will be included on my report to the authors and, if the manuscript is accepted for publication, my named report including any attachments I upload will be posted on the website along with the authors' responses. I agree for my report to be made available under an Open Access Creative Commons CC-BY license (http://creativecommons.org/licenses/by/4.0/). I understand that any comments which I do not wish to be included in my named report can be included as confidential comments to the editors, which will not be published.

Choose an item.

To further support our reviewers, we have joined with Publons, where you can gain additional credit to further highlight your hard work (see: https://publons.com/journal/530/gigascience). On publication of this paper, your review will be automatically added to Publons, you can then choose whether or not to claim your Publons credit. I understand this statement.

Yes Choose an item.
